# Adiponectin enhances bone marrow mesenchymal stem cell resistance to flow shear stress through AMP-activated protein kinase signaling

**DOI:** 10.1038/srep28752

**Published:** 2016-07-15

**Authors:** Lin Zhao, Chongxi Fan, Yu Zhang, Yang Yang, Dongjin Wang, Chao Deng, Wei Hu, Zhiqiang Ma, Shuai Jiang, Shouyi Di, Zhigang Qin, Jianjun Lv, Yang Sun, Wei Yi

**Affiliations:** 1 Department of Cardiovascular Surgery, Xijing Hospital, The Fourth Military Medical University, 127 Changle West Road, Xi’an 710032, China; 2 Department of Geriatrics, Xijing Hospital, The Fourth Military Medical University, 127 Changle West Road, Xi’an 710032, China; 3 Department of Thoracic Surgery, Tangdu Hospital, The Fourth Military Medical University, 1 Xinsi Road, Xi’an 710038, China; 4 Department of Thoracic and Cardiovascular Surgery, Affiliated Drum Tower Hospital of Nanjing University Medical School, 321 Zhongshan Road, Nanjing 210008, Jiangsu, China; 5 Department of Biomedical Engineering, The Fourth Military Medical University, 169 Changle West Road, Xi’an 710032, China; 6 Department of Aerospace Medicine, The Fourth Military Medical University, Xi’an 710032, China

## Abstract

Adiponectin has been demonstrated to protect the cardiovascular system and bone marrow mesenchymal stem cells (BMSCs). However, it is unclear whether adiponectin can protect BMSCs against flow shear stress (FSS). In this study, our aim was to explore the effects of adiponectin on BMSCs and to explore the role of AMP-activated protein kinase (AMPK) signaling in this process. Shear stress significantly inhibits the survival and increases the apoptosis of BMSCs in an intensity-dependent manner. The expression levels of TGF-β, bFGF, VEGF, PDGF, and Bcl2 are simultaneously reduced, and the phosphorylation levels of AMPK and ACC, as well as the expression level of Bax, are increased. Supplementation with adiponectin promotes the survival of BMSCs; reverses the changes in the expression levels of TGF-β, bFGF, VEGF, PDGF, Bcl2, and Bax; and further amplifies the phosphorylation of AMPK and ACC. Furthermore, the protective effects of adiponectin can be partially neutralized by AMPK siRNA. In summary, we have demonstrated for the first time that adiponectin can effectively protect BMSCs from FSS and that this effect depends, at least in part, on the activation of AMPK signaling.

Valvular heart disease (VHD) refers to the structural and functional disorders of the valves and is a common and growing problem in clinics^1^. In industrialized countries, the prevalence of VHD is approximately 2.5 percent, most cases of which are attributed to aortic stenosis and mitral regurgitation^2^, and in the United States, VHD accounts for a significantly increasing number of deaths in the aging population^3^. Furthermore, the conditions are worse in developing countries, where rheumatic heart disease remains the leading cause of VHD^2^. Artificial heart valve replacement has become the most effective treatment for VHD, which replaces the native valves with mechanical or bioprosthetic valves, thereby prolonging the lifespan of patients with VHD^4^,^5^. However, prosthetic valves are not flawless. Mechanical valves are durable but are more prone to thrombosis, and patients require lifelong anticoagulant therapy, which in turn increases the risk of hemorrhage. In contrast, bioprosthetic valves have outstanding hemodynamic performance but are degraded and calcified more easily^4^,^5^. Additionally, the inability to grow with pediatric patients is an even greater limitation of these prosthetic valves^6^. As a result, autologous tissue-engineered heart valves (TEHVs) have become the most attractive replacement valves because they can overcome the limitations of mechanical and bioprosthetic valves with their ability to remodel, regenerate, and grow^6^,^7^. To engineer heart valves, harvested cells are seeded onto decellularized valvular scaffolds to generate a tissue-engineered construct *in vitro*. They are then implanted into the diseased heart^8^. The seeded cells used to construct the TEHVs mainly include adipose mesenchymal stem cells, endothelial progenitor cells, and bone marrow mesenchymal stem cells (BMSCs)^8^,^9^,^10^. However, the current TEHVs do not adapt well to high shear stress when transplanted *in vivo*^11^. Therefore, there is a need to enhance the resistance of seeded cells to flow shear stress.

Adiponectin (APN, also known as adipocyte complement-related protein of 30 kD, adipoQ, apM1, and GBP28) is an adipokine secreted by adipose tissues and other cells, including cardiomyocytes^12^, whose expression levels are negatively correlated with cerebrovascular, cardiovascular and metabolic diseases, indicating an important role of adiponectin in the cardiovascular system^13^,^14^,^15^,^16^. Adiponectin exhibits protective effects on various cellular processes, including energy metabolism, inflammation, and proliferation, performing anti-hyperglycemic, anti-inflammatory, and anti-atherogenic functions^17^. In particular, adiponectin maintains myocardial cell survival, attenuates ischemia reperfusion injury (IRI), and protects the heart against pressure overload-induced dysfunction, as well as structural and metabolic remodeling^18^,^19^,^20^. Therefore, we speculated that adiponectin has a protective effect on BMSCs, whereby it increases the attachment of BMSCs to decellularized heart valve scaffolds, as well as increases the resistance of TEHVs to flow shear stress.

Adenosine monophosphate (AMP)-activated protein kinase (AMPK) is a serine/threonine protein kinase with high conservation in evolution that is involved in the regulation of cellular energy status^21^ by regulating the phosphorylation state of its substrates, especially acetyl CoA carboxylase (ACC)^22^. Its expression exerts a variety of effects on multiple tissues and organs, such as the liver, brain, skeletal muscle, and heart^23^,^24^,^25^. The effects of AMPK activation include the metabolic regulation of glucose, cholesterol, and fatty acids^26^, as well as cell growth, apoptosis, and autophagy^27^. Importantly, it has been reported that the endogenous and exogenous activation of AMPK plays a role in heart protection, including the prevention of myocardial ischemic injury^28^, cardiac fibrosis^21^, and heart failure^29^, as well as protection against cardiac pressure overload^30^,^31^. Additionally, studies have shown that adiponectin can activate the AMPK-dependent signaling pathway, exerting its anti-IRI and anti-pressure overload actions^18^,^20^,^32^. Therefore, we hypothesized that the effects of adiponectin on maintaining the attachment of BMSCs to decellularized heart valve scaffolds and enhancing the resistance to flow shear stress are mediated by AMPK signaling.

This study was designed to investigate the effects of adiponectin on the activity and function of BMSCs to facilitate their adaption to FSS (FSS). Furthermore, we aimed to explore the underlying mechanism of AMPK signaling in the resistance of BMSCs to flow shear stress induced by adiponectin, contributing to the development of TEHVs against high pressure and flow *in vivo*.

## Results

### Characterization of cultured rat BMSCs

**Figure 1:**
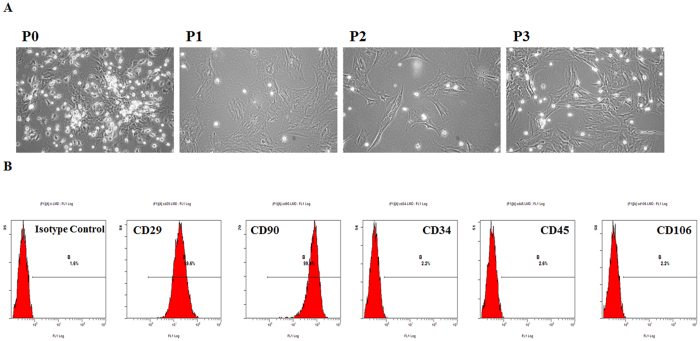
Morphology and phenotype of rat BM-MSCs. (**A**) Rat BMSCs showed homogenous, fibroblast-like morphology especially at the third passage. (**B**) Fluorescence-activated cell sorting (FACS) analysis of immune markers in rat BMSCs. The results confirmed that rat BMSCs were positive for CD29 and CD90 but negative for CD34, CD45, and CD106.

BMSCs were isolated and expanded from SD rats. Most cultured adherent cells showed the fibroblastic morphology that is characteristic of MSCs, particularly in the third-generation cells (Fig. 1A). In addition, FACS analysis demonstrated that BMSCs were 99.4% pure for CD90 and 99.6% pure for CD29. The percentages of contaminated populations of hematopoietic stem cells positive for CD34, CD45, and CD106 were 2.2%, 2.6%, and 2.2%, respectively (Fig. 1B).

### Effect of FSS on the cellular metabolic viability and apoptosis of rat BMSCs

**Figure 2:**
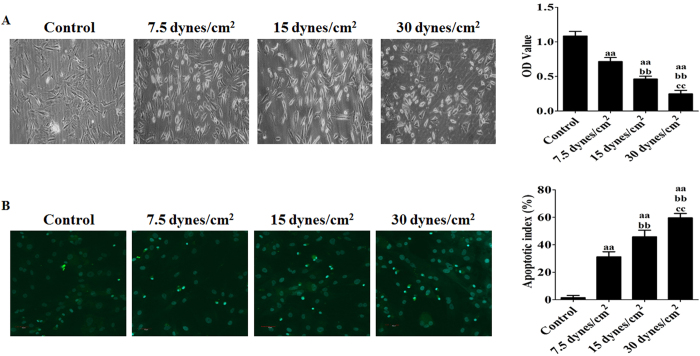
Effect of FSS on the cellular metabolic viability and apoptosis of rat BMSCs. (**A**) Representative morphology of rat BMSCs was demonstrated following exposure to different intensities of FSS (0, 7.5, 15, or 30 dynes/cm^2^) for 24 h. (**B**) Apoptotic index of rat BMSCs subjected to FSS injury is shown. Apoptotic cells were visualized by green fluorescence. All data are presented as fold changes vs. the control. The results are expressed as the mean ± SD, n = 6. ^aa^P < 0.05 vs. the control group; ^bb^P < 0.05 vs. the 7.5 dynes/cm^2^ FSS-treated group; ^cc^P < 0.05 vs. the 15 dynes/cm^2^ FSS-treated group.

Four different magnitudes of FSS (0, 7.5, 15, and 30 dynes/cm^2^) were applied to the MSCs for 24 h. After being seeded in 96-well plates for 24 h, the cellular metabolic viability was measured by the CCK-8 assay after being further cultured for 2 h in a humidified atmosphere of 5% CO_2_ at 37 °C. The data are shown in Fig. 2A. Compared with the control group (without FSS), stimulation of BMSCs for 24 h with 7.5, 15, or 30 dynes/cm^2^ FSS inhibited cell viability in an intensity-dependent manner (P < 0.05; Fig. 2A, right). Microscopy images (Fig. 2A, left) indicated that the FSS stimulation resulted in significant cell shrinkage and reduced the rate of cellular attachment compared with the control group.

The apoptotic index of FSS-treated rat BMSCs was also measured by a TUNEL assay. After stimulation with 7.5, 15, or 30 dynes/cm^2^ FSS for 24 h, the apoptotic index (Fig. 2B) increased from (1.56 ± 1.60)% to (31.28 ± 3.65)%, (45.76 ± 4.81)%, and (59.62 ± 3.14)%, respectively (P < 0.05), showing an intensity-dependent increase in apoptosis induction.

### Effects of FSS stimulation on local bFGF, TGF-β, VEGF, and PDGF levels in rat BMSCs

**Figure 3:**
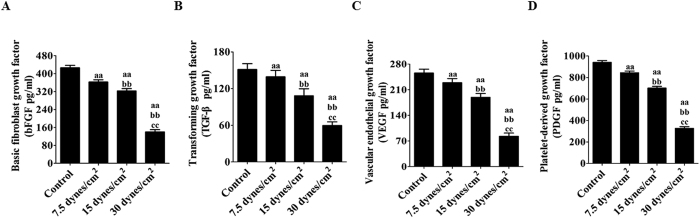
Effects of FSS stimulation on local production of bFGF, TGF-β, VEGF, and PDGF levels in rat BMSCs. Differences in cytokine levels in BMSC lysates. Productions of these cytokines were measured by an enzyme-linked immunosorbent assay (ELISA). All data are presented as fold changes vs. the control. (**A**) Levels of bFGF. (**B**) Levels of TGF-β. (**C**) Levels of VEGF. (**D**) Levels of PDGF. The results are expressed as the mean ± SD, n = 6. ^aa^P < 0.05 vs. the control group; ^bb^P < 0.05 vs. the 7.5 dynes/cm^2^ FSS-treated group; ^cc^P < 0.05 vs. the 15 dynes/cm^2^ FSS-treated group.

To measure the levels of various trophic factors, ELISAs were used to analyze the protein supernatants obtained from the BMSC cultures from each group. As shown in Fig. 3, the levels of bFGF, TGF-β, VEGF, and PDGF differed between the groups stimulated with FSS (7.5, 15, and 30 dynes/cm^2^) for 24 h. Additionally, the bFGF level was reduced in BMSCs from (426 ± 10.61)% to (363 ± 9.88)%, (323 ± 10.42)%, and (140 ± 9.89)% following FSS stimulation at 7.5, 15, and 30 dynes/cm^2^, respectively (P < 0.05, Fig. 3A), while TGF-β levels were significantly decreased from (151 ± 9.57)% to (139 ± 10.62)%, (108 ± 11.51)%, and (59.6 ± 5.88)% after FSS stimulation (P < 0.05, Fig. 3B). VEGF and PDGF have been reported to function as the ligands for PDGFR activation in MSCs^33^. After stimulation with different intensities of FSS, VEGF and PDGF levels changed in a similar manner from VEGF levels of (256 ± 10.44)% to (229 ± 11.29)%, (189 ± 10.26)%, and (82.9 ± 8.66)% (P < 0.05, Fig. 3C) and from PDGF levels of (941 ± 16.94)% to (844 ± 15.88)%, (705 ± 16.46)%, and (327 ± 15.51)%, respectively (P < 0.05, Fig. 3D).

### Effects of FSS stimulation on AMPK signaling and the Bcl2 protein family in rat BMSCs

**Figure 4:**
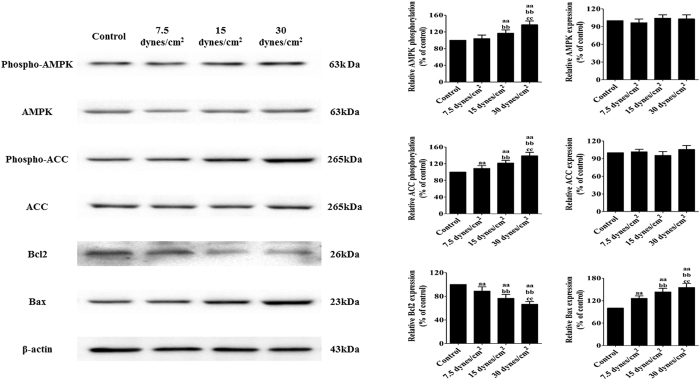
Effects of FSS stimulation on AMPK signaling and the Bcl2 protein family in rat BMSCs. BMSCs were stimulated with different intensities of FSS (0, 7.5, 15, or 30 dynes/cm^2^) for 24 h. Cell lysates were collected as described in the Materials and Methods section and subjected to Western blotting analysis with antibodies specific to p-AMPK, AMPK, p-ACC, ACC, Bcl2, Bax, and β-actin; β-actin served as the control. The statistics are representative of bands for the control and treatment groups. The results are expressed as the mean ± SD, n = 6. ^aa^P < 0.05 vs. the control group; ^bb^P < 0.05 vs. the 7.5 dynes/cm^2^ FSS-treated group; ^cc^P < 0.05 vs. the 15 dynes/cm^2^ FSS-treated group.

Activation of the AMPK pathway plays an important role in the regulation of BMSCs in many diseases^34^,^35^. To investigate the role of AMPK signaling in FSS stimulation-induced BMSC injury, AMPK-related molecules were detected by Western blotting. As shown in Fig. 4, FSS stimulations at different intensities induced a dose-dependent up-regulation of the phosphorylation of AMPK (P < 0.05). As a downstream target of AMPK, ACC phosphorylation was also examined. After being stimulated with FSS, the phosphorylation of ACC was similarly enhanced (P < 0.05). Additionally, FSS stimulation decreased the expression of Bcl2 and increased the expression of Bax (P < 0.05).

### APN promoted the cellular viability of and reduced apoptosis in FSS-cultured rat BMSCs

**Figure 5:**
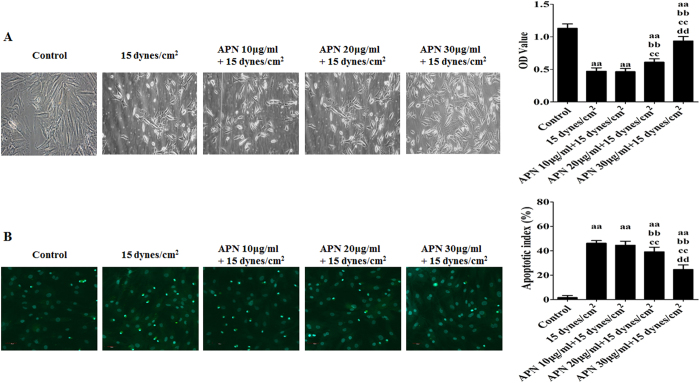
APN promoted the cellular viability of and reduced apoptosis in FSS-cultured rat BMSCs. BMSCs were exposed to the FSS environment (15 dynes/cm^2^) with or without adiponectin pretreatment at different concentrations (10, 20, and 30 μg/mL) for 18 h. The cellular viability and apoptotic index were assessed as described in the Materials and Methods section. (**A**) Representative morphology of rat BMSCs. (**B**) Apoptotic index of rat BMSCs. Apoptotic cells were visualized by green fluorescence. All data are presented as fold changes vs. the control. The results are expressed as the mean ± SD, n = 6. ^aa^P < 0.05 vs. the control group; ^bb^P < 0.05 vs. the 15 dynes/cm^2^ FSS-treated group; ^cc^P < 0.05 vs. the 15 dynes/cm^2^ FSS-treated + 10 μg/mL APN group; ^dd^P < 0.05 vs. the 15 dynes/cm^2^ FSS-treated + 20 μg/mL APN group.

To determine the effect of APN on the cellular viability of and apoptosis in rat BMSCs, the cells were exposed to the FSS environment with or without adiponectin pretreatment at different concentrations (10, 20, and 30 μg/mL) for 18 h^36^. In the experimental groups, BMSCs were chosen to undergo treatment with 15 dynes/cm^2^ FSS for 24 h because they were too severely damaged following treatment with 30 dynes/cm^2^ FSS according to our above trial (Fig. 2A). The cellular viability and apoptotic index of rat BMSCs were assessed by CCK-8 analysis and the TUNEL assay. The APN-treated BMSCs exhibited better survival than untreated MSCs under FSS stimulation, especially at the 30-μg/mL concentration (P < 0.05; Fig. 5A, right). Microscopy images (Fig. 5A, left) indicated that the APN treatments significantly ameliorated cell shrinkage and increased the rate of cellular attachment induced by FSS.

Additionally, the apoptotic index of APN-pretreated BMSCs exposed to the FSS environment was also measured. After pre-treatments with 10, 20, and 30 μg/mL APN for 18 h, the apoptotic index induced by FSS was reduced from (46.21 ± 2.14)% to (44.58 ± 3.27)%, (39.15 ± 3.83)%, and (24.66 ± 4.01)%, respectively (P < 0.05, Fig. 5B), demonstrating a dose-dependent decrease in apoptosis induction.

### APN restores the local production levels of bFGF, TGF-β, VEGF, and PDGF in FSS-cultured rat BMSCs

**Figure 6:**
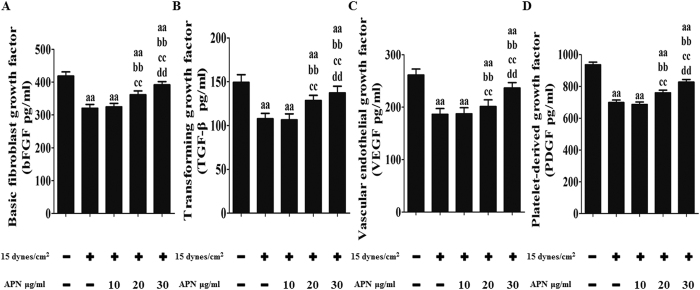
APN restored the local production of bFGF, TGF-β, VEGF, and PDGF in FSS-cultured rat BMSCs. BMSCs were exposed to the FSS environment (15 dynes/cm^2^) with or without adiponectin pretreatment at different concentrations (10, 20, and 30 μg/mL) for 18 h. The cytokines, such as bFGF, TGF-β, VEGF, and PDGF, were assessed as described in the Materials and Methods section. All data are presented as fold changes vs. the control. (**A**) Levels of bFGF. (**B**) Levels of TGF-β. (**C**) Levels of VEGF. (**D**) Levels of PDGF. The results are expressed as the mean ± SD, n = 6. ^aa^P < 0.05 vs. the control group; ^bb^P < 0.05 vs. the 15 dynes/cm^2^ FSS-treated group; ^cc^P < 0.05 vs. the 15 dynes/cm^2^ FSS-treated + 10 μg/mL APN group; ^dd^P < 0.05 vs. the 15 dynes/cm^2^ FSS-treated + 20 μg/mL APN group.

The above experiments demonstrated the reductions of cytokines in BMSCs mediated by FSS injury. Next, the protective effect of adiponectin on cytokines in BMSCs subjected to FSS injury was examined in a similar manner. As demonstrated in Fig. 6A–D, pre-treatment with APN (10, 20, or 30 μg/mL) for 18 h resulted in a dose-dependent increase in bFGF production following FSS-induced injury, from (321 ± 11.49)% to (325 ± 10.61)%, (362 ± 11.65)%, and (394 ± 9.88)%, respectively (P < 0.05, Fig. 6A). The TGF-β level was also significantly increased from (108 ± 6.21)% to (106 ± 6.87)%, (129 ± 5.88)%, and (137 ± 7.68)%, respectively, after APN pre-treatment (P < 0.05, Fig. 6B). Furthermore, after APN administration (10, 20, and 30 μg/mL) for 18 h, VEGF and PDGF levels recovered in a similar manner from (186 ± 10.91)% to (186 ± 11.67)%, (201 ± 12.55)%, and (236 ± 10.25)% for VEGF (P < 0.05, Fig. 6C) and from (698 ± 17.01)% to (687 ± 15.77)%, (760 ± 15.21)%, and (827 ± 16.42)% for PDGF (P < 0.05, Fig. 6D).

### APN pre-treatment modulates AMPK signaling and the Bcl2 protein family in FSS-cultured rat BMSCs

**Figure 7:**
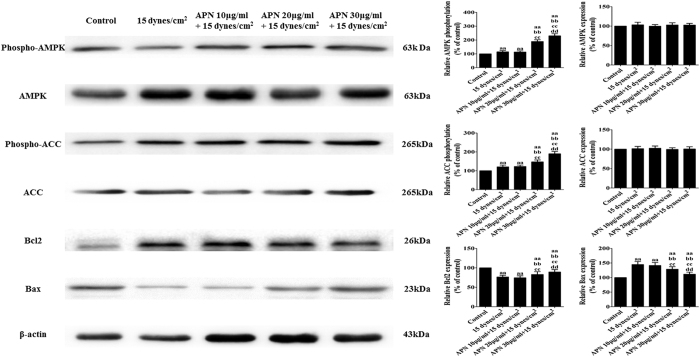
APN pre-treatment modulated AMPK signaling and the Bcl2 protein family in FSS-cultured rat BMSCs. BMSCs were exposed to the FSS environment (15 dynes/cm^2^) with or without adiponectin pretreatment at different concentrations (10, 20, and 30 μg/mL) for 18 h. Cell lysates were collected as described in the Materials and Methods section and subjected to Western blotting analysis with antibodies specific to p-AMPK, AMPK, p-ACC, ACC, Bcl2, Bax, and β-actin; β-actin served as the control. The statistics are representative of bands corresponding to the control and treatment groups. The results are expressed as the mean ± SD, n = 6. ^aa^P < 0.05 vs. the control group; ^bb^P < 0.05 vs. the 15 dynes/cm^2^ FSS-treated group; ^cc^P < 0.05 vs. the 15 dynes/cm^2^ FSS-treated + 10 μg/mL APN group; ^dd^P < 0.05 vs. the 15 dynes/cm^2^ FSS-treated + 20 μg/mL APN group.

To assess the impact of the APN pre-treatment on signaling pathways activated in FSS-cultured rat BMSCs, the expression and activity of AMPK signaling and the expression of the Bcl2 protein family were compared between the groups. APN pre-treatment further increased the phosphorylation of AMPK and ACC expression, which were altered in FSS-injured BMSCs (Fig. 7). Additionally, APN pre-treatment increased the expression of Bcl2 and decreased the expression of Bax (P < 0.05).

### APN protects against FSS-induced changes in cellular viability and cytokines in rat BMSCs via AMPK

**Figure 8:**
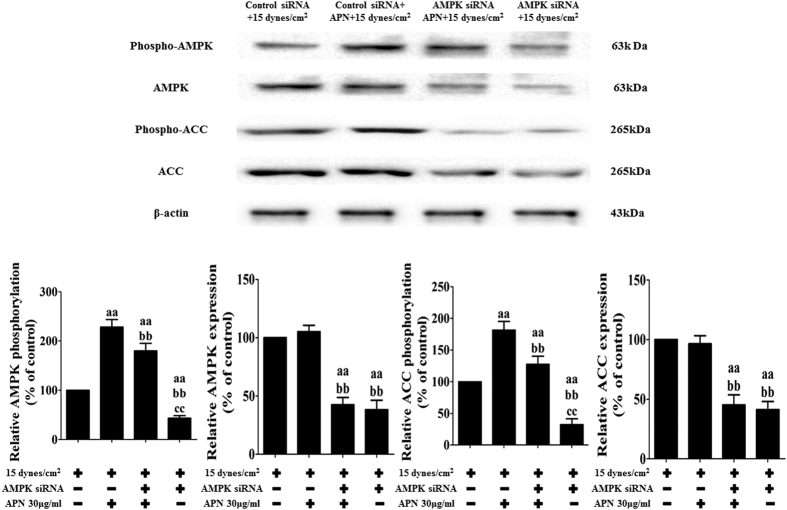
AMPK siRNA inhibited the expression of its downstream proteins. BMSCs were transfected with siRNA targeting AMPK or nonspecific scramble siRNA (control siRNA) for 48 h and then treated with APN (30 μg/mL) for 18 h, followed by exposure to FSS (15 dynes/cm^2^) for 24 h. Cell lysates were collected as described in the Materials and Methods section and subjected to Western blotting analysis with antibodies specific to p-AMPK, AMPK, p-ACC, and ACC; AMPK and ACC served as controls. The statistics are representative of bands corresponding to the control and treatment groups. The results are expressed as the mean ± SD, n = 6. ^aa^P < 0.05 vs. the control siRNA + 15 dynes/cm^2^ FSS-treated group; ^bb^P < 0.05 vs. the control siRNA+ 30 μg/mL APN + 15 dynes/cm^2^ FSS-treated group; ^cc^P < 0.05 vs. the AMPK siRNA+ 30 μg/mL APN + 15 dynes/cm^2^ FSS-treated group.

**Figure 9:**
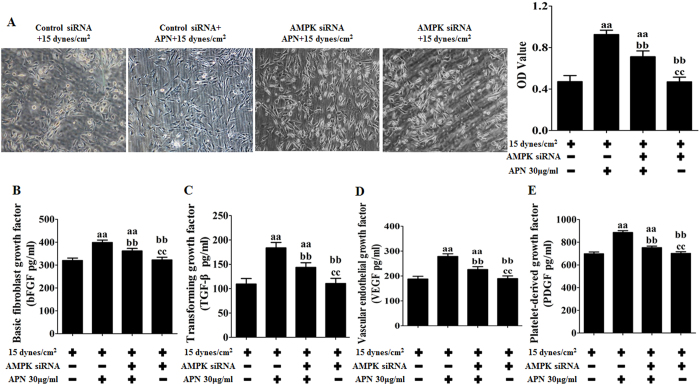
APN protected against FSS-induced changes in cellular viability and cytokines in rat BMSCs via AMPK. BMSCs were transfected with siRNA targeting AMPK or nonspecific scramble siRNA (control siRNA) for 48 h and then treated with APN (30 μg/mL) for 18 h, followed by exposure to FSS (15 dynes/cm^2^) for 24 h. The cellular viability and cytokine levels were assessed as described previously. (**A**) Cell viability and representative morphology of rat BMSCs. (**B**) Levels of bFGF. (**C**) Levels of TGF-β. (**D**) Levels of VEGF. (**E**) Levels of PDGF. The results are expressed as the mean ± SD, n = 6. ^aa^P < 0.05 vs. the control siRNA + 15 dynes/cm^2^ FSS-treated group; ^bb^P < 0.05 vs. the control siRNA + 30 μg/mL APN + 15 dynes/cm^2^ FSS-treated group; ^cc^P < 0.05 vs. the AMPK siRNA + 30 μg/mL APN + 15 dynes/cm^2^ FSS-treated group.

To investigate the role of AMPK on the protective effects of APN, AMPK was silenced by siRNA, followed by APN (30 μg/mL) treatment for 18 h and FSS (15 dynes/cm^2^) for an additional 24 h. AMPK siRNA did not affect the cell viability and cytokines (P > 0.05 compared with the control group, Supplementary Fig. 1). However, AMPK siRNA transfection significantly attenuated the APN-induced up-regulation of AMPK (P < 0.05 compared with the control siRNA + APN + FSS group, Fig. 8). Furthermore, silencing AMPK reversed the protective effects of APN against FSS-induced BMSC death (P < 0.05 compared with the control siRNA + FSS group, Fig. 9A). Inhibition of AMPK by siRNA also reduced the productions of cytokines (P < 0.05 compared with the control siRNA + APN + FSS group, Fig. 9B), such as bFGF, TGF-β, VEGF, and PDGF, in rat BMSCs.

## Discussion

VHD is a common and growing problem world-wide^2^,^3^. Artificial heart valve replacement has become the most effective treatment for VHD to prolong the lifespan and improve the life quality of patients^4^,^5^. However, there are problems that limit the utilization of prosthetic and mechanical valves^4^,^5^,^6^. TEHVs have attracted the most attention in valve replacement^6^,^7^. Additionally, adipose mesenchymal stem cells, endothelial progenitor cells, and BMSCs are often used^8^,^9^,^10^. However, the current TEHVs do not adapt well to high shear stress when transplanted *in vivo*^11^. Luo *et al*.^37^ found that FSS caused a dose-related reduction of the proliferation rate of MSCs, with the majority of cells being arrested in the G0 or G1 phase *in vitro*. Using confocal microscopy, Cartmell and colleagues^38^ also observed that a high flow rate of 1.0 mL/min resulted in substantial cell death throughout the constructs after one week of culture. Other study has also demonstrated that a high flow velocity significantly affected cell morphology, cell-cell interactions, matrix production, and matrix composition^39^. In our study, we also found that the apoptosis of BMSCs increases with the amplification of shear stress, which can be dose-dependently attenuated by APN.

TGF-β, bFGF, VEGF, and PDGF are important growth factors involved in healing and are markedly up-regulated following tendon injury^40^. Furthermore, they are also important in the development of robust TEHVs^41^,^42^,^43^. Kural *et al*.^41^ found that isometric cell force, passive retraction, and collagen production can be tuned by independently altering boundary stiffness and TGF-β1 concentration. The ability to stimulate matrix production without inducing high active tension will aid in the development of robust TEHVs and other connective tissue replacements, where minimizing tissue shrinkage upon implantation is critical. In particular, the combination of TGF-β1 with EGF and PDGF induces vascular smooth muscle cell proliferation and expression of extracellular matrix constituents found in the aortic valve^42^. In this study, we found that TGF-β, bFGF, VEGF, and PDGF levels can be decreased by shear stress in an intensity-dependent manner. However, these phenomena can be dose-dependently inhibited by APN.

APN is an adipokine secreted by adipose tissues and other cells^12^ and has demonstrated protective roles in the cardiovascular system^13^,^14^,^15^. The protection afforded by APN from myocardial ischemia-reperfusion injury has been widely demonstrated^18^,^44^. APN can also inhibit the structural and metabolic remodeling of the heart. During pressure overload, APN deficiency accelerated the transition from cardiac hypertrophy to heart failure^20^ and cardiac fibrosis^36^, but pretreatment with recombinant APN significantly decreased H_2_O_2_-induced cardiomyocyte hypertrophy by decreasing total protein levels, protein synthesis, and atrial natriuretic factor and brain natriuretic peptide expression^36^. In doxorubicin-induced cardiomyopathy, APN also improved cardiac function through anti-apoptotic effects^45^. More importantly, APN infusion ameliorated diabetic mobilopathy of BMSCs and contributed to the mobilization and recruitment of BMSCs to participate in tissue repair and regeneration^46^. However, whether APN can protect BMSCs in the development of TEHVs is still unclear. In the current study, we observed that APN contributes to the survival of and apoptosis inhibition in BMSCs and neutralizes the influence of FSS on BMSCs in TEHVs.

Previous studies have demonstrated that the activation of AMPK contributes to the protection of the heart against myocardial ischemic injury^28^, cardiac fibrosis^21^, heart failure^29^, and cardiac pressure overload^30^,^31^. Additionally, the primary biological effects of APN are induced by the stimulation of AdipoR1 and AdipoR2, which increase the activities of 5′AMP and the peroxisome proliferator-activated receptor, respectively, and stimulate AMPK phosphorylation and uncoupling protein 2 up-regulation^47^,^48^,^49^. In particular, numerous studies have shown that through the activation of the AMPK-dependent signaling pathway, APN exerts anti-IRI, anti-pressure overload, and anti-apoptotic actions^18^,^20^,^32^,^45^. ACC is an important substrate of APN, and the activation of AMPK can further promote the phosphorylation of ACC^22^. In this study, we found that both AMPK and ACC phosphorylation are increased under FSS, which can be further amplified by APN. Bcl2 and Bax have been demonstrated to function as an apoptotic activator and apoptotic inhibitor, respectively. The activation of various molecular signals from AMPK can regulate the Bcl2/Bax apoptosis pathway^50^,^51^,^52^,^53^. In particular, Kim *et al*.^50^ reported that triggering the activation of AMPK by various factors resulted in an increase in Bax gene expression and promotion of apoptosis. Other study has also found that the activation of AMPK alpha-1 alleviates endothelial cell apoptosis by increasing the expression of Bcl-2^53^. Our results demonstrated that the activation of AMPK by APN can reverse the increased apoptosis of BMSCs by the upregulation of Bcl2 and downregulation of Bax. Furthermore, the AMPK siRNA can partially reverse the protective effect of APN against BMSCs by decreasing TGF-β, bFGF, VEGF, and PDGF expression levels and inhibiting ACC phosphorylation.

**Figure 10:**
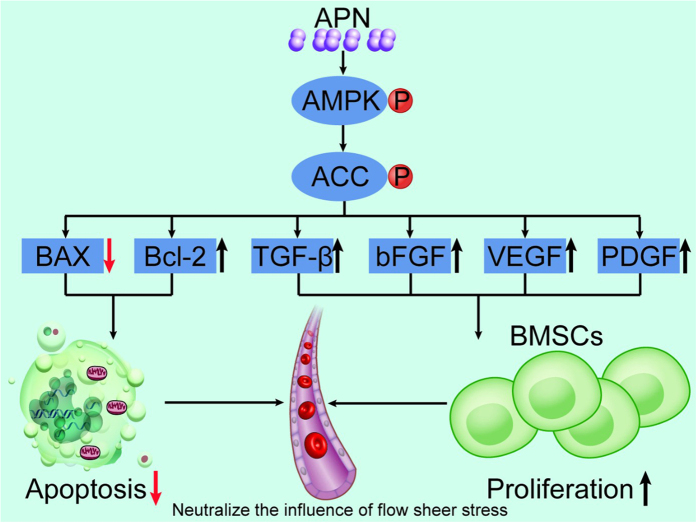
Adiponectin enhances bone marrow mesenchymal stem cell resistance to flow shear stress through AMP-activated protein kinase signaling. APN can effectively protect BMSCs from FSS during the development of TEHVs. Additionally, the protective effects of APN are attributed to the activation of AMPK, which can further promote ACC phosphorylation, Bcl2 up-regulation, and Bax down-regulation.

As shown in Fig. 10, we have demonstrated for the first time that APN can effectively protect BMSCs from FSS during the development of TEHVs. Additionally, the protective effects of APN are attributed to the activation of AMPK, which can further promote ACC phosphorylation, Bcl2 up-regulation, and Bax down-regulation.

## Methods and Materials

### Materials

Recombinant globular rat APN, dimethylsulfoxide (DMSO), 4′,6-diamino-2-phenylindole (DAPI), trypsin, and protease inhibitor cocktail were purchased from Sigma-Aldrich (St. Louis, MO, USA). Low-glucose Dulbecco’s modified Eagle’s medium (L-DMEM), antibiotic/antimycotic solution, and fetal bovine serum (FBS) were obtained from Gibco Laboratories (Life Technologies, Inc., Burlington, ON, Canada). The Cell Counting Kit-8 (CCK8) assay was purchased from Dojindo Molecular Technologies (Kumamoto, Japan). Terminal deoxynucleotidyl transferase dUTP nick-end labeling (TUNEL) kits were purchased from Roche (Mannheim, Germany). IgG Isotype Control and the FITC-conjugated anti-mouse CD29, CD45, and CD90 were purchased from eBioscience (San Diego, CA, USA), and CD34 and CD106 were purchased from Santa Cruz Biotechnology (Dallas, TX, USA). Antibodies against adenosine monophosphate (AMP)-activated protein kinase (AMPK), phospho-AMPK (p-AMPK), and β-actin were obtained from Cell Signaling Technology (Beverly, MA, USA). Antibodies against acetyl CoA carboxylase (ACC), phospho-ACC (p-ACC), Bcl2, and Bax were purchased from Santa Cruz Biotechnology (Santa Cruz, CA, USA). Rabbit anti-goat, goat anti-rabbit, and goat anti-mouse secondary antibodies were purchased from the Zhongshan Company (Beijing, China). The BCA assay kit was purchased from Pierce (Rockford, IL, USA). Scramble and AMPK siRNAs were obtained from Ambion (Austin, TX, USA). INTERFERin siRNA transfection reagent was purchased from Polyplus (New York, NY, USA). A bFGF ELISA kit was purchased from Cusabio Biotech (Wuhan, China). The PDGF ELISA kit was purchased from Quantikine (R&D Systems, Minneapolis, MN, Canada). TGF-b1 and VEGF ELISA kits were purchased from eBioscience (San Diego, CA, USA). Other reagents were all purchased from Sigma, unless otherwise specified.

### Animals and ethics

Two-month-old male Sprague-Dawley (S-D) rats, with body weights of 110–130 g, were bought from the Fourth Military Medical University Animal Center in China. Animals were bred in an environmentally controlled room (20–23 °C, 60% humidity, 12-hour light-dark cycle) and fed sterile water and standard laboratory rat chow ad libitum. All the procedures and experimental protocols were approved by the Fourth Military Medical University Animal Care and Use Committee and performed in accordance with the Health Guide for the Care and Use of Laboratory Animals of National Institutes.

### BMSC culture and expansion *in vitro*

The isolation and culture of BMSCs were performed as described previously^54^. In brief, after administering appropriate anesthesia, the bilateral femurs and tibias of SD rats were aseptically excised after removing connective tissues around the bones. Then, the femurs and tibiae were stored in phosphate-buffered saline (PBS) supplemented with 1% penicillin/streptomycin on ice, and the surfaces were further trimmed and cleaned. The bones were broken, and the bone marrow was flushed with L-DMEM containing 10% FBS and 1% penicillin/streptomycin. The fluid was collected into the beaker, and the dispersed bone marrow cells were centrifuged at 350× g for five minutes. Bone marrow cells were harvested and incubated with the same medium supplemented in a humidified atmosphere of 5% CO_2_ at 37 °C. The medium was changed for the first time after 48 h, with removal of non-adherent cells, and the media was subsequently changed once every three days. Once the cell fusion reached 80–90%, the cells were washed with PBS, trypsinized using 0.25% trypsin at 37 °C, and subcultured under the same conditions. Cultured BMSCs at the third passage were used for the following experiments.

### Fluorescence-activated cell sorting analysis of BMSCs

Cultured cells were subjected to fluorescence-activated cell sorting (FACS) analysis to assess the expression of various signature antigen markers of BMSCs^55^. Third-generation cells that exhibited good growth were selected to prepare single-cell suspensions. After being trypsinized with 0.25% trypsin, the cells were collected, centrifuged, rinsed, and re-suspended in PBS at a concentration of 10^5^ cells/mL. Re-suspended cells were incubated with 5 mL of fluorescein isothiocyanate-labeled anti-rat IgG Isotype Control, CD29, CD34, CD45, CD90, and CD106 in the dark at 4 °C for 30 minutes. After being washed twice with PBS, the cells were analyzed using a flow cytometer (BD FACSCanto™ II; BD Biosciences). The data were analyzed using CellQuest software (BD Biosciences). IgG Isotype Control and the FITC-conjugated anti-mouse, CD29, CD45, and CD90 antibodies were purchased from eBioscience (San Diego, USA); CD34 and CD106 were purchased from Santa Cruz Biotechnology (Dallas, TX, USA); and the others were purchased from Abcam (Cambridge, MA, USA).

### Shear stress stimulation and adiponectin treatments

Shear stress stimulation of BMSCs was carried out in a parallel plate flow chamber with a flow loop apparatus that was designed by Jacobs *et al*.^56^ and improved by Bai *et al*.^57^ in previous studies. The passage-3 BMSCs at 1.5 × 10^4^ cells/cm^2^ were trypsinized and cultured on the bottom of the parallel plate flow chamber (10.0 cm in length, 2.5 cm in width, and 0.03 cm in height) for one day before exposure to FSS. After the cells had adhered to the slide, the medium was driven by a constant hydrostatic pressure through the channel of the flow chamber, and cells were exposed to steady and well-defined FSS^58^. The FSS intensity (τ, dynes/cm^2^) was computed according to the formula τ = 6 μQ/wh^2^, where μ is the viscosity of the medium and Q is the flow volume. FSS stimulations were performed under the same culture conditions as those described above. A peristaltic pump was used to generate shear stress. The duration of FSS was 24 h.

Recombinant globular rat adiponectin was dissolved in L-DMEM containing 0.1% BSA to ensure a concentration of 1.0 mg/mL. This stock solution was stored at 4 °C for one week and diluted with culture medium immediately prior to the experiment. After pretreatment with FSS for 24 h, the cells were subjected to different concentrations of APN (10, 20, and 30 μg/mL) for 18 h. The morphology was then assessed under an inverted/phase contrast microscope, and images were obtained using a 600D camera (Canon Company, Japan). Finally, the cells were harvested for further analysis.

### Cell proliferation analysis

Cell proliferation analysis was carried out as described in a previous study^59^. First, BMSCs were cultured with different processing methods. Then, cells from each group were seeded into 96-well plates with 100 μL of cell suspension (8,000 cells/well), with five wells for each group at each time point. After incubation at 37 °C for 24 h, the culture supernatant was removed and a total of 100 μL of serum-free medium and 10 μL of CCK-8 solution were added to each well. After incubation at 37 °C with 5% CO_2_ for 2 h, the optical density (OD) values were measured at 450 nm using a microplate reader (SpectraMax 190, Molecular Devices, USA), and cell viability was expressed as the OD value.

### Cell apoptosis assay

Apoptosis was assessed using the terminal deoxynucleotidyl transferase-mediated deoxyuridine triphosphate nick-end labeling (TUNEL) assay^60^. BMSCs were first plated onto cover slips at a density of 5 × 10^4^ cells/well in 24-well plates. After different treatments, the cover slips with cells were treated with 3% hydrogen peroxide diluted in methanol for 15 minutes to block endogenous peroxidase activity and then fixed in 4% paraformaldehyde for 30 minutes at 4 °C. The cells were then incubated in 0.1% Triton X-100 for 20 minutes on ice. After washing with PBS, the cells were covered with 50 μL of the TUNEL reaction mixture and two negative controls using 50 μL of label solution per control. Next, all of the cell samples were incubated in this solution for 60 minutes at 37 °C in a dark humidified chamber. Finally, the cells were stained with DAPI. The apoptotic cells were visually identified in ten selected fields and photographed at a magnification of ×200 under an Olympus FV1000 (Olympus, Japan) confocal microscope.

### Enzyme-linked immunosorbent assay

The bFGF, TGF-b, VEGF, and PDGF concentrations were measured using an enzyme-linked immunosorbent assay (ELISA). For this assay, the treated BMSCs were collected and washed with ice-cold PBS. After 48 h, culture supernatants were divided into 200-μL samples in triplicate. Finally, these samples were homogenized in pre-chilled lysis buffer (20 mM HEPES, 0.5 mM EGTA, 1 mM DTT, and 0.32 M sucrose; PH 7.4) containing protease inhibitors cocktail and then incubated at 4 °C for 30 minutes. The samples were centrifuged at 15, 000 rpm in a pre-chilled centrifuge at 4 °C for ten minutes. The supernatant from the cell lysate was collected and assayed for protein concentration using the Pierce™ BCA Protein Assay reagent kit (Thermo Fisher Scientific, Waltham, MA, USA). Samples containing equal amounts of total proteins were analyzed by ELISA kits for bFGF, TGF-β, VEGF, and PDGF according to the manufacturers’ protocols. The sensitivity of these kits was less than 1 ng/mL, the intra-assay coefficient of variation was 10%, and the inter-assay coefficient of variation was 10%. All the reported results are based on three independent experiments on separate batches of cells.

### Western blot analysis

Samples were lysed in detergent lysis buffer (20 mM Tris-HCl, pH 7.5; 150 mM NaCl; 1 mM EDTA; 1 mM EGTA; 1% Triton; 2.5 mM sodium pyrophosphate; 1 mM β-glycerophosphate; 1 mM Na_3_VO_4_; and 1 μg/mL leupeptin). The lysates were centrifuged at 12, 000 rpm for 25 minutes at 4 °C, and protein concentrations were measured using the Pierce™ BCA Protein Assay reagent kit (Thermo Fisher Scientific). Ten micrograms of protein were separated by 7.5–10% sodium dodecyl sulfate-polyacrylamide gel electrophoresis (SDS-PAGE) (Bio-Rad, Hercules, CA, USA), and separated proteins were electrophoretically transferred onto polyvinylidene difluoride (PVDF) membranes (Millipore, Billerica, MA). Membranes were blocked with Tween-20 Tris-buffered saline (10 mM Tris-HCl, pH 7.5; 100 mM NaCl; and 0.1% Tween 20) containing 5% nonfat dry milk before incubation with rabbit monoclonal anti-adenosine monophosphate (AMP)-activated protein kinase (AMPK) antibody (1:1,000), rabbit monoclonal anti-phospho-AMPK (p-AMPK) antibody (1:1,000), goat polyclonal anti-acetyl CoA carboxylase (ACC) antibody (1:500), rabbit polyclonal anti-phospho-ACC (p-ACC) antibody (1:500), goat polyclonal anti-Bcl2 antibody (1:500), rabbit polyclonal anti-Bax antibody (1:500), and rabbit monoclonal anti-β-actin antibody (1:1,000). Antigen detection was performed using enhanced chemiluminescence Western blotting detection reagents (Amersham Pharmacia Biotech, Piscataway, NJ, USA) with horseradish peroxidase-conjugated secondary antibodies. Images were obtained and densities were determined using Image J. The protein expression was corrected by the β-actin density, and the expression in the control group was arbitrarily set at 100%.

### Small interfering RNA transfection

Utilizing specific AMPK RNA, interference was performed against the AMPK target sequence (5′-AAGAGAAGCAGAAGCACGACG-3′)^61^. A nonspecific scramble siRNA was used as the control. Cells were seeded into six-well plates. After the cells reached 70–80% confluence, they were transfected with AMPK siRNA or NC siRNA. Transfections were performed using Lipofectamine 2000 transfection reagent (Invitrogen, Carlsbad, CA, USA) according to the manufacturer’s instructions with 100 nM of each siRNA^62^. The efficiency of siRNA-mediated AMPK knockdown was verified by Western blot analysis 48 h after transfection. Twenty-four hours after transfection, the cells were treated with APN (30 μg/mL) for 18 h and FSS (15 dynes/cm^2^) for 24 h. Finally, the cells were collected and processed for subsequent use in further experiments.

### Statistical analysis

All of the values are presented as the mean ± standard deviation (SD). Group comparisons were carried out using ANOVA (SPSS 15.0, SPSS Inc., Chicago, IL, USA). All of the groups were analyzed simultaneously using one-way ANOVA with the Bonferroni correction for multiple comparisons. A P value less than 0.05 was regarded as a statistically significant difference.

## Additional Information

**How to cite this article**: Zhao, L. *et al*. Adiponectin enhances bone marrow mesenchymal stem cell resistance to flow shear stress through AMP-activated protein kinase signaling. *Sci. Rep*. **6**, 28752; doi: 10.1038/srep28752 (2016).

## Supplementary Material

Supplementary Information
