# Supplementary material for: Adiponectin enhances bone marrow mesenchymal stem cell resistance to flow shear stress through AMP-activated protein kinase signaling
Source: Sci Rep. 2016 Jul 15;6:28752. doi: 10.1038/srep28752 (PMC4945870; doi:10.1038/srep28752)

Supplementary Information

Title of manuscript:

Adiponectin enhances bone marrow mesenchymal stem cell resistance to flow shear stress through AMP-activated protein kinase signaling

Authors:

Lin Zhao, Chongxi Fan, Yu Zhang, Yang Yang, Dongjin Wang, Chao Deng, Wei Hu, Zhiqiang Ma, Shuai Jiang, Shouyi Di, Zhigang Qin, Jianjun Lv, Yang Sun, and Wei Yi

Supplementary information includes:

Supplementary Figs. S1-S4

Supplementary Fig. 1 Effects of Control siRNA on cell viability and local production of bFGF, TGF-β, VEGF, and PDGF levels in rat BMSCs. BMSCs were transfected with nonspecific scramble siRNA (control siRNA) for 48 h. The cellular viability and productions of cytokines (bFGF, TGF-β, PDGF, and VEGF) were assessed as described in the Materials and Methods section. **A.** Representative morphology of rat BMSCs. **B.** cell viability statistical graph. **C.** Local production of cytokines (bFGF, TGF-β, VEGF, and PDGF). The results are expressed as the mean ± SD, n=6.

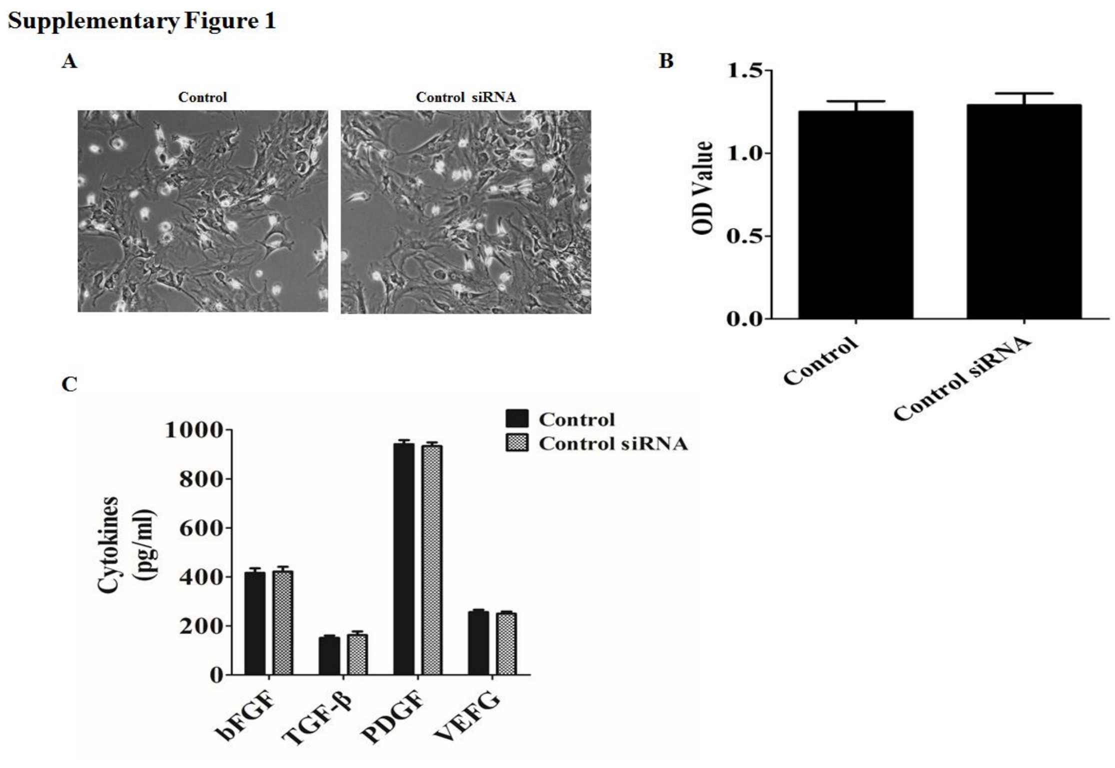

Supplementary Fig. 2: The full length blots in Fig. 4

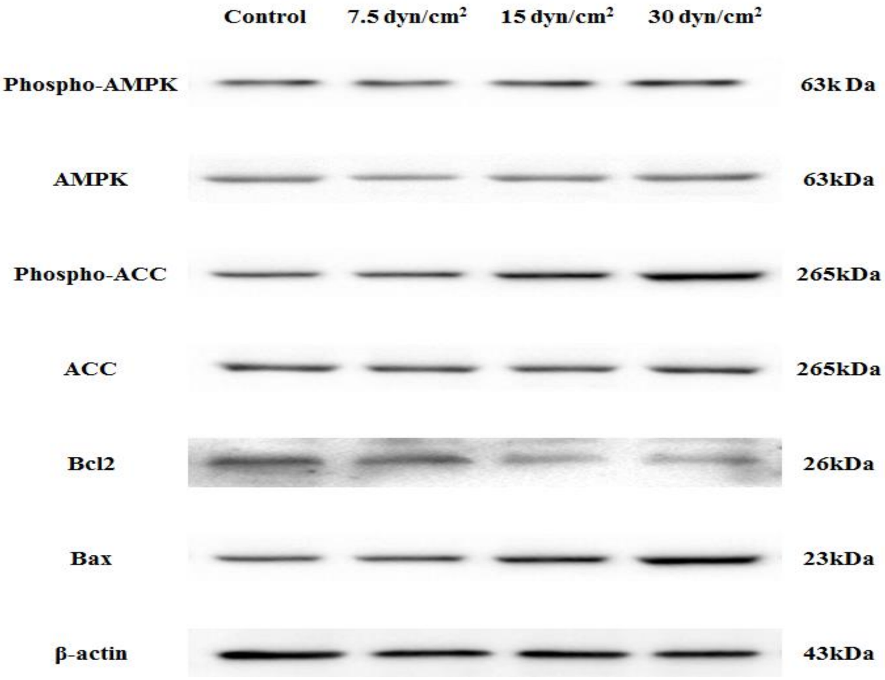

Supplementary Fig. 3: The full length blots in Fig. 7

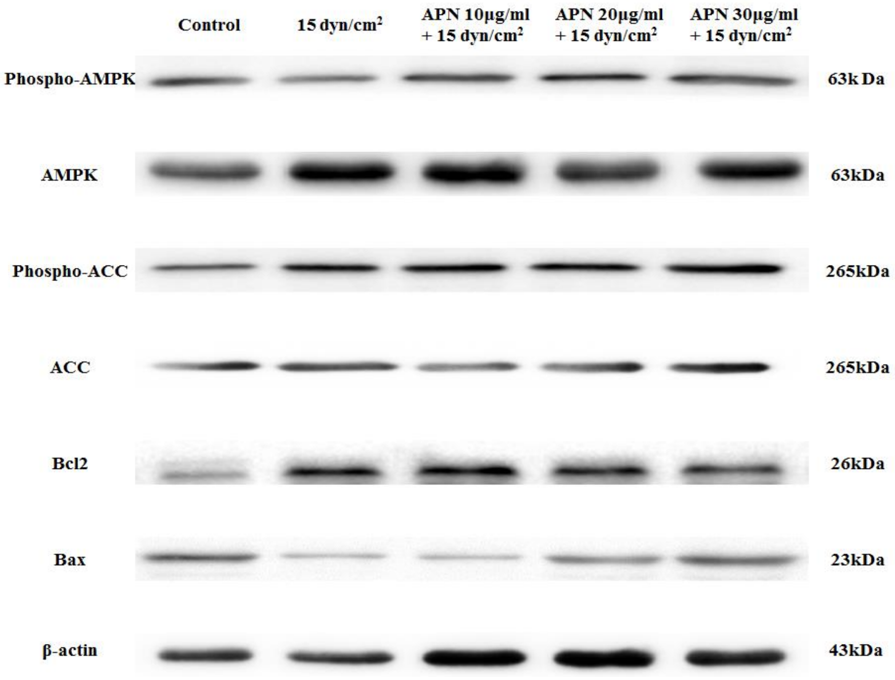

Supplementary Fig. 4: The full length blots in Fig. 8

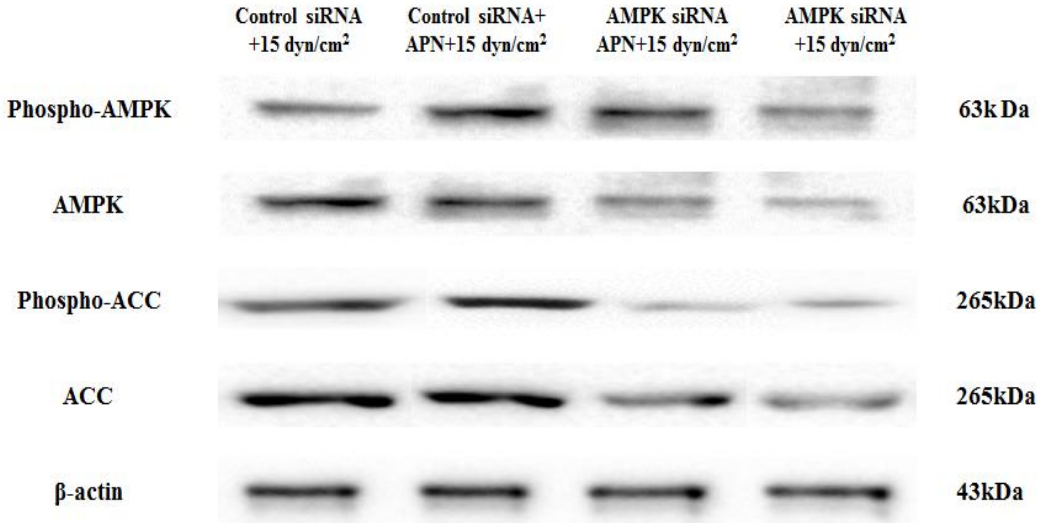

Supplement: Supplementary Information [file srep28752-s1.pdf]
